# Silencing of a BAHD acyltransferase in sugarcane increases biomass digestibility

**DOI:** 10.1186/s13068-019-1450-7

**Published:** 2019-05-06

**Authors:** Wagner Rodrigo de Souza, Thályta Fraga Pacheco, Karoline Estefani Duarte, Bruno Leite Sampaio, Patrícia Abrão de Oliveira Molinari, Polyana Kelly Martins, Thaís Ribeiro Santiago, Eduardo Fernandes Formighieri, Felipe Vinecky, Ana Paula Ribeiro, Bárbara Andrade Dias Brito da Cunha, Adilson Kenji Kobayashi, Rowan Andrew Craig Mitchell, Dasciana de Sousa Rodrigues Gambetta, Hugo Bruno Correa Molinari

**Affiliations:** 1 Genetics and Biotechnology Laboratory, Embrapa Agroenergy (CNPAE), Brasília, DF 70770-901 Brazil; 2 Centre of Natural Sciences and Humanities, Federal University of ABC, São Bernardo do Campo, SP 09606-045 Brazil; 3 Plant Sciences, Rothamsted Research, Harpenden, Hertfordshire AL5 2JQ UK

**Keywords:** Sugarcane, Cell-wall acylation, Biomass, Lignocellulosic feedstock, Biofuels

## Abstract

**Background:**

Sugarcane (*Saccharum* spp.) covers vast areas of land (around 25 million ha worldwide), and its processing is already linked into infrastructure for producing bioethanol in many countries. This makes it an ideal candidate for improving composition of its residues (mostly cell walls), making them more suitable for cellulosic ethanol production. In this paper, we report an approach to improving saccharification of sugarcane straw by RNAi silencing of the recently discovered *BAHD01* gene responsible for feruloylation of grass cell walls.

**Results:**

We identified six *BAHD* genes in the sugarcane genome (Sac*BAHD*s) and generated five lines with substantially decreased *SacBAHD01* expression. To find optimal conditions for determining saccharification of sugarcane straw, we tried multiple combinations of solvent and temperature pretreatment conditions, devising a predictive model for finding their effects on glucose release. Under optimal conditions, demonstrated by *Organosolv* pretreatment using 30% ethanol for 240 min, transgenic lines showed increases in saccharification efficiency of up to 24%. The three lines with improved saccharification efficiency had lower cell-wall ferulate content but unchanged monosaccharide and lignin compositions.

**Conclusions:**

The silencing of *SacBAHD01* gene and subsequent decrease of cell-wall ferulate contents indicate a promising novel biotechnological approach for improving the suitability of sugarcane residues for cellulosic ethanol production. In addition, the *Organosolv* pretreatment of the genetically modified biomass and the optimal conditions for the enzymatic hydrolysis presented here might be incorporated in the sugarcane industry for bioethanol production.

**Electronic supplementary material:**

The online version of this article (10.1186/s13068-019-1450-7) contains supplementary material, which is available to authorized users.

## Background

Lignocellulosic biomass is among the most abundant biological resources on Earth. This biomass is produced as a primary product for animal feed or as residues from food crops and other agroindustrial wastes. Digestibility of the lignocellulosic biomass, which is the ease with which sugars can be released from the cell-wall polysaccharides, is a key economic target. The improvement of biomass digestibility may lead to the increased production of biofuels and other value-added products and to a better efficiency of digestion by ruminant animals [1].

Sugarcane (*Saccharum* spp.) is a semiperennial grass crop mainly grown for sugar and ethanol production. The processing of sugarcane generates two major biomass residues: the fibrous fraction following juice extraction from the stalks (bagasse); and the harvest residue, consisting of green tops and older leaves, known as straw [2, 3]. Both biomasses can be used as feedstock to generate heat and electricity and to produce cellulosic ethanol. In addition, the sugarcane straw is commonly used to improve soil quality [3]. The sugarcane processing can generate between 7.4 and 20 Mg ha^−1^ of straw in dry matter, but may reach up to 30 Mg ha^−1^ year^−1^ when high-yield sugarcane plants are used [4–6]. These large volumes of straw can be left on the field, where it will naturally decompose to act as a soil fertilizer, or it can be harvested to generate bioenergy. The utilization of the straw should occupy a prominent place as a feedstock for the production of cellulosic ethanol. Some studies suggest that one ton of straw corresponds to 1.2–2.8 barrel of oil equivalent [7, 8]. Increasing the efficiency and yield of this sugarcane straw conversion to bioethanol (as well as bagasse) is a major target to increase economic viability of bioethanol making it more competitive compared to fossil fuels.

The major barrier that hinders the use of plant biomass for biofuel production is the recalcitrance of the plant cell wall. In general, plant cell walls are composed of cellulosic microfibrils embedded in a matrix of hemicelluloses, pectins, lignin, and proteins, but the specific composition of cell walls depends on the plant species. In grass cell walls, the major hemicellulosic polysaccharide is xylan, which is commonly substituted with arabinofuranose (Ara*f*) and ester-linked hydroxycinnamates. In addition, the β-(1-4)-xylose backbone can be substituted with α-(1,2)-glucuronic acid to form glucuronoarabinoxylans (GAX), which contains both arabinosyl and glucuronosyl residues on the β-1,4 xylan backbone [9]. Another distinctive feature of grass cell walls is the prevalence of two hydroxycinnamates: *p*-coumarate (*p*CA) and ferulate (FA) [10]. FA is involved in grass cell-wall crosslinking reactions through the acylation of arabinofuranosyl units that are (1-3)-linked to the xylan backbone in arabinoxylan (AX) or GAX. Ester-linked FA is coupled oxidatively in a similar manner to that of lignin monomers [11, 12], forming crosslinks with other (G)AX chains or with lignin [13–16]. This complex network of crosslinks in grass cell walls can inhibit the digestion by preventing enzymatic access to the biomass, making the cell-wall deconstruction and the release of fermentable sugars for ethanol production difficult. Therefore, decreasing FA content and consequently FA-mediated crosslinks of grass biomass has long been considered a promising strategy for increasing digestibility [1, 17].

Although the precise mechanisms by which FA is incorporated into AX are not fully elucidated, the genes responsible for this phenomenon in grasses are starting to emerge. In this sense, genes belonging to the BAHD acyl-coenzyme A (CoA) transferase superfamily are being designated as possible candidates for feruloylation of AX in grass cell walls [18]. Some studies suggested the role of BAHD genes in AX feruloylation [19–21], but the strongest evidence of the involvement of a BAHD gene in FA incorporation was demonstrated recently by [1]. Using *Setaria viridis*, an emerging plant model for grasses [22], those authors showed that suppression of *SvBAHD01*, a member of the BAHD acyl-CoA transferase gene family in *S. viridis*, was able to reduce up to 60% the levels of AX feruloylation, drastically increasing the saccharification of the biomass [1]. Therefore, silencing of *SvBAHD01* orthologs may be a suitable strategy for the improvement of biomass digestibility in other species.

In industrial processes, it is essential to provide accessibility of the cellulose in the biomass to hydrolytic enzymes, to release fermentable sugars for ethanol production. This is mainly achieved by (*i*) using biomass feedstocks that possess less cell-wall recalcitrance; (*ii*) using suitable pretreatments of the raw material for hemicellulose sugars extraction; and (*iii*) enzymatic treatment for the cellulose conversion to glucose that will be converted to ethanol by fermentation [23]. Among the pretreatment technologies, which include *Organosolv* treatment [24], acid and alkali pretreatments [25, 26], and steam explosion [27], the *Organosolv* process has been considered as one of the most promising for cellulosic ethanol production [23, 24, 28, 29]. This type of pretreatment involves the use of an organic liquid and water to partially hydrolyze lignin bonds and lignin-carbohydrate bonds, resulting in a solid residue consisting of mainly cellulose and some hemicellulose [30, 31]. There are many advantages that emerge from the use of *Organosolv* pretreatment in industrial processes, mainly because of its low cost and ease of recovery, miscibility in water, and low toxicity. Therefore, the use of suitable biomass feedstocks followed by a feasible and cost-effective pretreatment and implementing enzymatic hydrolysis processes are the major bottlenecks for cellulosic ethanol production on the industrial scale.

In this study, we identify the BAHD acyl-CoA transferase gene family and describe the effects of silencing *BAHD01* gene expression in a commercial sugarcane cultivar. We show that sugarcane BAHD01 RNAi lines have improved straw saccharification. In addition, we developed a predictive model for the *Organosolv* pretreatment and enzymatic hydrolysis analysis of sugarcane straw biomass, designed to find the optimal conditions for the screening of a large number of transgenic events.

## Results

### Identification of BAHD acyl-CoA gene family members and generation of silencing lines in sugarcane

**Fig. 1:**
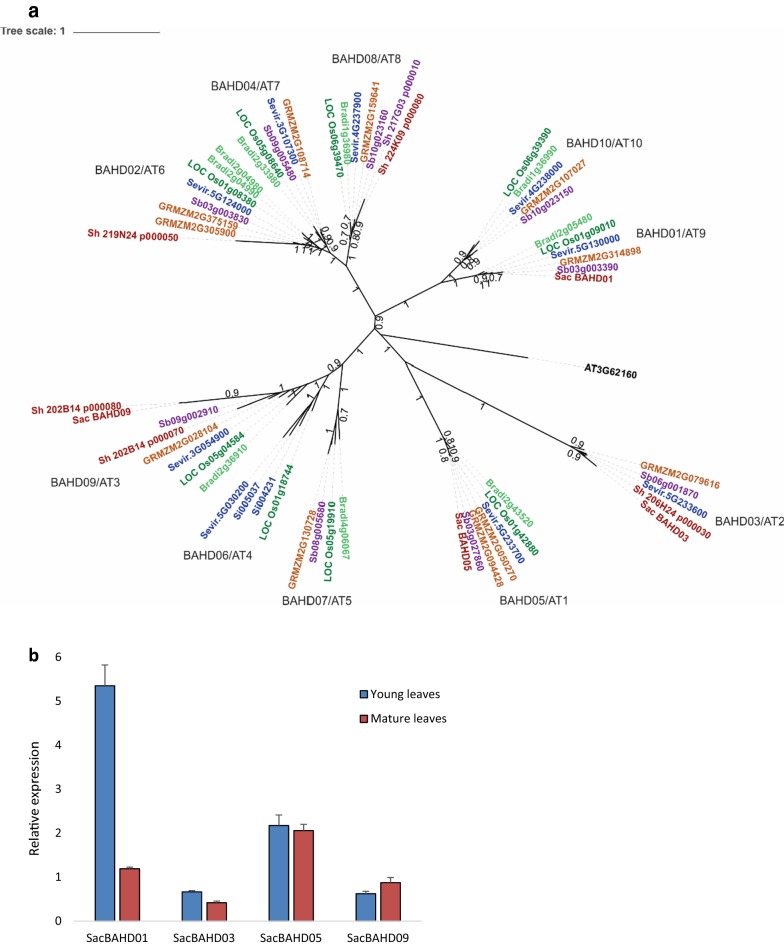
Phylogenetic and expression analyses of candidate clade *BAHD* genes. **a** Phylogenetic tree of candidate *BAHD* genes identified in Arabidopsis (AT), *Brachypodium* (Bradi), maize (GRMZM), rice (LOC Os), sorghum (Sb), *Setaria viridis (*Sevir)*, Setaria italic* (Si), and sugarcane (Sac, Riacho-Panõn and Matiello [32]; Sh, Garsmeur et al. [33]). Support for the topology is shown as fraction of bootstrap runs. BAHD names for each branch are based on Molinari et al. [46], and alternative AT names are based on Bartley et al. [20]. **b** Real-time qPCR analysis of the identified *BAHD* genes in sugarcane. Expression is relative to the high expressed reference genes *GAPDH* and *EF1*-*α*. Young and mature leaves correspond to tissues from three- and eight-month-old sugarcane plants, respectively (*n* = 5; error bars ± SEM)

To identify *BAHD01* gene in the sugarcane genome [32], we analyzed the phylogeny of *BAHD* genes in the ‘Mitchell Clade’ [20] for sugarcane, *Setaria*, *Brachypodium*, maize, rice, and Arabidopsis (Fig. 1a). We were able to identify four *BAHD* genes in the sugarcane genome, named *SacBAHD01*, *SacBAHD03*, *SacBAHD05*, and *SacBAHD09* based on the nomenclature suggested for *Setaria viridis* BAHD genes [1]. It is important to mention that the selection of gene targets and transformation was initiated before the release of a more complete sugarcane genome [33]. However, to generate the phylogenetic tree presented in Fig. 1a, we also identified BAHD homologs based on this new released genome. The BAHD homologs identified in the genome released by Riacho-Panõn and Matiello [32] and Gasmeur et al. [33] are identified in the phylogenetic tree as “Sac” and “Sh,” respectively. From these genes, Sac*BAHD01* and *SacBAHD05* presented the highest levels of expression in sugarcane leaves collected from 3- and 8-month-old plants, which were classified as young and mature leaves, respectively (Fig. 1b). *SacBAHD01* was highly expressed in young leaves of sugarcane, decreasing its expression as the leaves mature, while *SacBAHD05* transcript levels did not change significantly during different developmental stages.

To suppress the expression of *SacBAHD01*, a construct was designed to contain an RNAi hairpin under control of a constitutive maize ubiquitin promoter (Additional file 1: Figure S1a). RNAi BAHD01 matches 100% to a specific region of *SacBAHD01* CDS sequence (Additional file 1: Figure S1b), and by comparing the sequence of this region with other BAHD gene sequences found in both sugarcane genomes [32, 33], we could verify the specificity of our RNAi BAHD01 sequence. The RNAi target sequence was formed from a 413 bp region toward the 3′ end of the CDS of SAC_BAHD01 (825-1237 bp). The longest stretch of identity with off-target BAHD genes included 24 bp (SAC_BAHD10*), 16 bp (SAC_BAHD02*), 15 bp (SAC_BAHD05), 14 bp (SAC_BAHD03, SAC_BAHD08*), 13 bp (SAC_BAHD09), 10 bp (SAC_BAHD04*), and < 10 bp (SAC_BAHD7*). Sequences marked with * were estimated from the database used rather than full-length cDNA clones. Therefore only SAC_BAHD10 has sufficient length of identity to possibly match a few short interfering RNAs produced from the hairpin compared with hundreds that would match SAC_BAHD01. Furthermore, two SNPs present in this 24 bp stretch in SAC_BAHD10 suggest that only one of several paralogs of SAC_BAHD10 actually match the RNAi. However, the *SacBAHD10* gene was not expressed significantly in young and mature leaves of wild-type sugarcane SP80-3280 and did not show any compensation of its expression levels in transgenic plants, as demonstrated by RNA sequencing analysis (de Souza et al. [1]; manuscript in preparation).

SP80-3280 sugarcane variety was transformed with this construct, generating 14 independent events presenting different levels of *SacBAHD01* suppression (Additional file 2: Figure S2a). We selected five of the most suppressed lines (*SacBAHD01* RNAi lines 1, 2.2, 2.4, 3.1, and 4), which demonstrated silencing levels ranging from 70 to 95% (Additional file 2: Figure S2b) to perform biomass pretreatment and digestibility analysis.

### *Organosolv* pretreatment and saccharification analysis of SacBAHD01 RNAi lines

The physical or chemical pretreatment of the biomass is pivotal to provide broad access to cellulose by hydrolytic enzymes in industrial processes. To screen for the biomasses from *SacBAHD01* RNAi lines with improved digestibility, we decided on *Organosolv* pretreatment with ethanol as organic solvent, followed by enzymatic hydrolysis. We first established optimal enzymatic concentration to use on pretreated samples of wild-type plants by varying levels of a commercial mixture of cellulases and hemicellulases (Cellic^®^ CTec 3; Novozymes), and demonstrating that 15 FPU/g was the minimum enzymatic activity necessary for the maximum glucose release after 48 h (Additional file 3: Figure S3). Next, we evaluated and optimized the effects of two pretreatment variables—the ethanol concentration (X1) and residence time (*X*2) on biomass enzymatic saccharification—using the Response Surface Methodology (RSM) based on five-level Central Composite Design (CCD), originally described by Box and Wilson [34] and successfully used for optimizing processes [35]. We used sugarcane leaves and stem tops of 12-month-old plants (straw) from transgenic lines that displayed the highest levels of *SacBAHD01* suppression (events 1, 2.2, 2.4, 3.1, and 4) and nontransformed (NT) plants as biomass for *Organosolv* pretreatments.

Eleven experiments, under nine pretreatment conditions, were applied, according to experimental design, with ethanol concentration ranging from 30 to 100% (v/v) and residence time ranging from 0 to 240 min (Table 1). The pretreated samples were further submitted to enzymatic hydrolysis conducted in 6 and 48 h, at 15 FPU/g dry biomass. The different ethanol concentrations (X1) and the residence times (*X*2) of *Organosolv* treatments, in addition to the obtained glucose concentrations released after 6 and 48 h of saccharification of sugarcane biomass, are shown in Table 1. The difference between the responses of the experiments 9, 10, and 11 is due to an uncontrolled step such as temperature control in the reactor, for example, and has to be considered for the estimation of the model’s quality.

**Table 1:** Real and coded levels of variables used in CCD experiments and the responses glucose concentrations obtained with 6- and 48-h enzymatic hydrolysis ± SD

Trials	X1 Ethanol concentration (% v/v)	X2 Residence time (min)	NT	Ev. 1	Ev. 2.2	Ev. 2.4	Ev. 3.1	Ev. 4
Glucose 6 h (g L^−1^)								
1	− 1.0 (40.2)	− 1.0 (34.9)	13.48 ± 0.16^b^	13.30 ± 0.66^b^	14.06 ± 0.82^ab^	15.11 ± 0.11^a^	14.75 ± 0.26^a^	11.95 ± 0.32^c^
2	− 1.4 (30.0)	+ 1.0 (205.1)	21.75 ± 1.08^a^	21.04 ± 0.76^ab^	20.12 ± 0.05^ab^	20.27 ± 0.25^ab^	19.37 ± 0.70^ab^	18.79 ± 0.86^b^
3	+ 1.0 (89.8)	− 1.0 (34.9)	7.91 ± 0.15^ab^	7.37 ± 0.28^b^	8.30 ± 0.35^ab^	8.24 ± 0.32^a^	8.03 ± 0.50^ab^	7.43 ± 0.22^b^
4	+ 1.0 (89.8)	+ 1.0 (205.1)	10.15 ± 0.37^a^	9.94 ± 0.28^a^	10.15 ± 0.31^a^	10.53 ± 0.44^a^	10.18 ± 0.43^a^	9.77 ± 0.18^a^
5	− 1.4 (30.0)	0 (120.0)	21.08 ± 0.46^ab^	21.84 ± 0.87^a^	21.35 ± 0.62^ab^	20.05 ± 0.71^b^	21.05 ± 0.92^ab^	19.86 ± 0.41^b^
6	+ 1.4 (100.0)	0 (120.0)	6.26 ± 0.13^c^	6.19 ± 0.18^c^	7.20 ± 0.30^a^	6.95 ± 0.36^ab^	6.49 ± 0.10^bc^	6.95 ± 0.06^ab^
7	0 (65.0)	− 1.4 (0.0)	7.44 ± 0.30^bc^	7.42 ± 0.15^bc^	8.28 ± 0.49^a^	8.00 ± 0.33^ab^	7.61 ± 0.19^abc^	7.12 ± 0.29^c^
8	0 (65.0)	+ 1.4 (240.0)	16.89 ± 0.57^ab^	16.96 ± 1.04^ab^	15.44 ± 0.56^b^	17.93 ± 1.06^a^	17.94 ± 0.58^a^	16.47 ± 0.34^ab^
9	0 (65.0)	0 (120.0)	16.22 ± 0.67^a^	15.56 ± 0.35^a^	15.11 ± 0.73^a^	16.18 ± 0.90^a^	15.36 ± 0.87^a^	16.12 ± 0.79^a^
10	0 (65.0)	0 (120.0)	13.02 ± 0.37^bc^	13.15 ± 0.23^bc^	13.58 ± 0.23^ab^	14.62 ± 0.65^a^	12.28 ± 0.09 ^cd^	12.05 ± 0.58^d^
11	0 (65.0)	0 (120.0)	10.95 ± 0.51^b^	12.65 ± 0.12^a^	10.56 ± 0.47^b^	13.56 ± 0.68^a^	12.62 ± 0.29^a^	10.76 ± 0.58^b^
Glucose 48 h (g L^−1^)								
1	− 1.0 (40.2)	− 1.0 (34.9)	23.07 ± 0.46^b^	20.42 ± 0.77^bc^	22.07 ± 0.38^bc^	26.85 ± 1.56^a^	26.62 ± 2.80^a^	19.68 ± 1.24^c^
2	− 1.4 (30.0)	+ 1.0 (205.1)	31.33 ± 0.86^c^	35.68 ± 2.58^a^	32.31 ± 0.55^b^	34.74 ± 0.75^a^	31.75 ± 0.04^c^	28.46 ± 1.45^d^
3	+ 1.0 (89.8)	− 1.0 (34.9)	8.92 ± 0.66^b^	9.09 ± 0.66^b^	10.65 ± 0.40^a^	9.53 ± 1.01^ab^	10.03 ± 0.75^ab^	9.29 ± 0.28^ab^
4	+ 1.0 (89.8)	+ 1.0 (205.1)	13.69 ± 1.38^a^	13.07 ± 0.39^a^	14.72 ± 1.19^a^	13.95 ± 0.56^a^	14.66 ± 1.00^a^	13.37 ± 0.88^a^
5	− 1.4 (30.0)	0 (120.0)	31.77 ± 0.65^bc^	38.00 ± 0.96^a^	33.93 ± 1.53^b^	33.49 ± 1.59^b^	29.62 ± 0.76^c^	31.74 ± 0.88^bc^
6	+ 1.4 (100.0)	0 (120.0)	8.49 ± 0.28^a^	8.51 ± 0.17^a^	8.93 ± 0.24^a^	9.31 ± 0.44^a^	8.63 ± 0.49^a^	8.44 ± 1.13^a^
7	0 (65.0)	− 1.4 (0.0)	8.74 ± 0.29^c^	8.74 ± 0.06^c^	10.28 ± 0.85^a^	9.94 ± 0.31^ab^	9.17 ± 0.10^bc^	8.76 ± 0.71^c^
8	0 (65.0)	+ 1.4 (240.0)	26.27 ± 0.23^ab^	29.33 ± 3.55^a^	22.39 ± 0.44^b^	29.01 ± 2.81^a^	28.71 ± 3.22^a^	24.97 ± 1.00^ab^
9	0 (65.0)	0 (120.0)	25.75 ± 2.74^a^	22.29 ± 2.23^a^	22.01 ± 2.71^a^	22.67 ± 1.63^a^	22.45 ± 3.09^a^	22.65 ± 4.00^a^
10	0 (65.0)	0 (120.0)	25.17 ± 0.28^a^	23.02 ± 1.78^ab^	24.23 ± 0.76^a^	24.89 ± 2.11^a^	22.66 ± 1.30^ab^	20.14 ± 0.44^b^
11	0 (65.0)	0 (120.0)	16.89 ± 1.53^c^	25.51 ± 0.63^a^	16.02 ± 0.87^c^	22.97 ± 0.88^b^	23.26 ± 1.39^ab^	15.57 ± 1.13^c^

Means followed by the same lower case letters in a line do not differ significantly by the Tukey test (*p* < 0.05). In this case, letters should be compared only between the rows, but not between different trials. For each experimental condition, presented in a table row, a Tukey test was performed. These results are from four independent replicates. Ev. refers to different transgenic events. NT: nontransformed plants

Based on the experimental results described above and using RSM, we were able to propose a second order model for the prediction of glucose concentration released after pretreatment of each biomass analyzed. The regression equations and correlations coefficients considering the complete model and coded values are presented in Additional file 4: Table S1. The evaluation of the variable coefficients demonstrated that the ethanol concentration (*X*1) and residence time (*X*2) in the *Organosolv* pretreatment strongly contributed to glucose release in 6 and 48 h of enzymatic hydrolysis. The correlation coefficients suggest that there are close agreements between the experimental results and the theoretical values predicted by the polynomial models (Additional file 4: Table S1).

Using the models as objective functions, it was demonstrated that the maximum glucose concentration released after hydrolysis for all transgenic biomasses was obtained for pretreatment variables of 30% (v/v) ethanol concentration (*X*1 = − 1.41) and residence time of 240 min (*X*2 = 1.41). For NT plants, the maximum glucose release was found for pretreatment variables of 30% (v/v) ethanol concentration (*X*1 = − 1.41) and residence time of 223.8 min (*X*2 = 1.22).

The analysis of variance (ANOVA) at 90% confidence level indicated that the models are statistically significant. Despite the difference between the responses of the experiments 9, 10, and 11, the lack of fit, associated with these repetitions, is not significant, suggesting that the regression might be used for predictive purposes. The value of pure error was small for all responses, which indicates good reproducibility of the obtained data. In this sense, the pretreatment variables *X*1 and *X*2 could be modified to predict and optimize the glucose concentration released after hydrolysis of the sugarcane straw in industrial processes. The results predicted by the models are in agreement with experimental values in all cases, which proves that the trial 11 deviation was an isolated case, but without prejudice to the models reliability.

To assure the feasibility and accuracy of the proposed models, a pretreatment validation experiment was performed, using the optimal *Organosolv* conditions found for transgenic biomass, i.e., 30% (v/v) ethanol concentration and 240 min of residence time. The enzymatic hydrolysis was performed under the same conditions described above, using 15 FPU of the enzymatic cocktail per gram biomass over 6 and 48 h. Experimental and predicted values of glucose release for each biomass under these conditions are shown in Table 2. As observed, the data demonstrated that experimental values for glucose release are in agreement with the predicted values, corroborating that the models proposed from RSM could reliably predict the dependent variables.

**Table 2:** Experimental ± SD and predicted values for 6- and 48-h enzymatic hydrolyses and the respective percent errors under the pretreatment validation conditions [30% (v/v) ethanol concentration and 240 min of residence time for pretreatment]

Biomass	Experimental	Predicted	Error (%)
Glucose 6 h (g L^−1^)			
NT	24.54 ± 0.89	26.77	8.4
Ev. 1	24.55 ± 1.42	26.19	6.3
Ev. 2.2	22.69 ± 0.66	24.78	8.4
Ev. 2.4	23.03 ± 0.35	23.40	1.6
Ev. 3.1	20.84 ± 0.18	24.36	14.5
Ev. 4	20.06 ± 0.65	23.43	14.4
Glucose 48 h (g L^−1^)			
NT	35.66 ± 0.93	35.41	0.7
Ev. 1	44.31 ± 0.59	44.60	0.7
Ev. 2.2	41.33 ± 1.23	38.04	8.7
Ev. 2.4	39.63 ± 1.03	39.63	0.0
Ev. 3.1	29.26 ± 0.70	35.05	16.5
Ev. 4	36.89 ± 0.91	35.69	3.4

*NT* control, nontransformed plants, *Ev.* independent sugarcane transgenic events

**Fig. 2:**
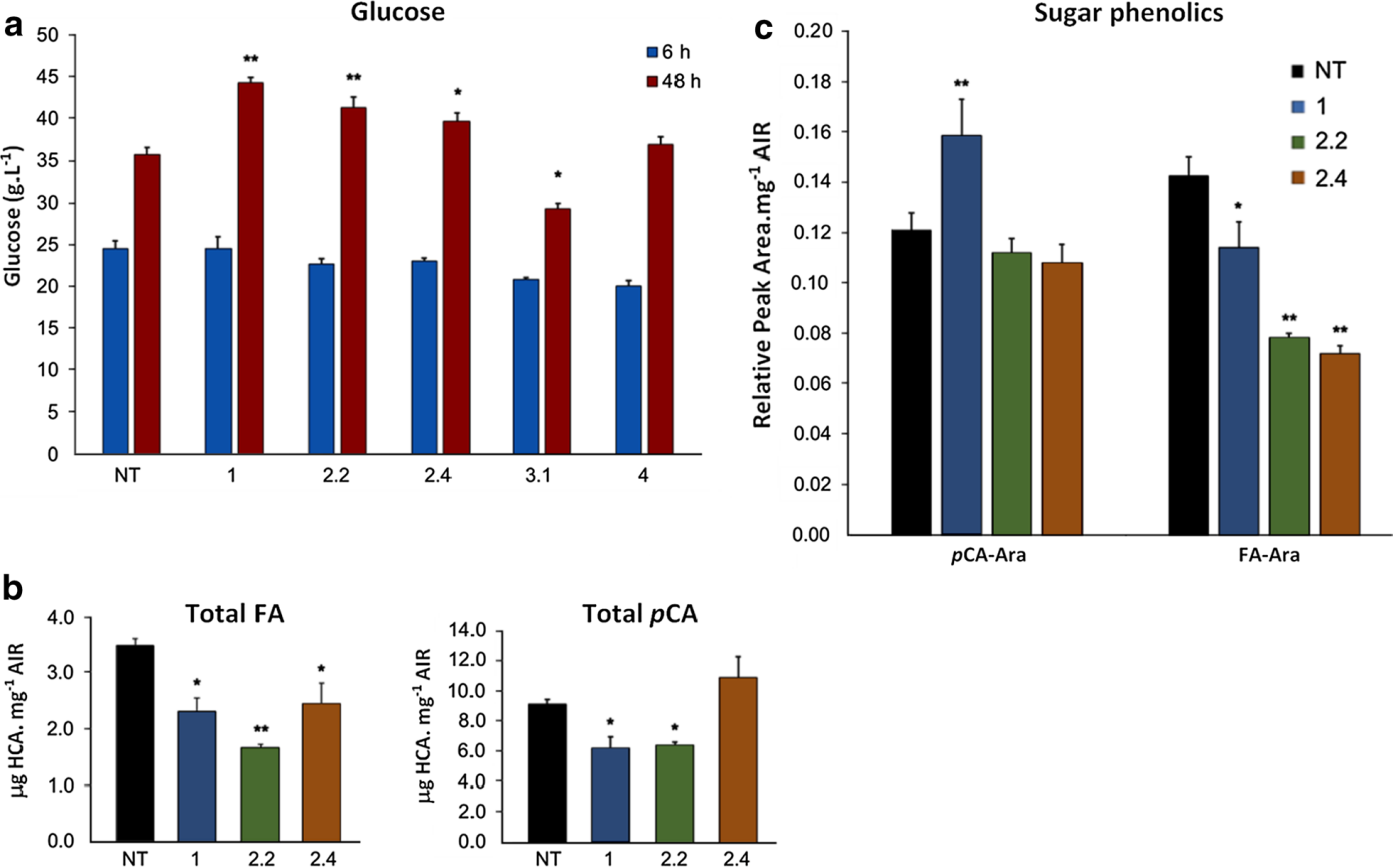
Cell-wall characterization and digestibility of sugarcane straw biomass. **a** Saccharification of sugarcane straw from SacBAHD01 RNAi lines and nontransformed (NT) plants after *Organosolv* pretreatment, using Cellic CTec 3 (Novozymes) at 15 FPU for 6 and 48 h. **b** Ester-linked contents of ferulate (FA) and *p*-coumarate acid (*p*CA) in the alcohol insoluble fraction (AIR) of sugarcane straw from three independent events of SacBAHD01 RNAi lines and NT plants. **c** HCA conjugates in supernatant following mild acidolysis of sugarcane straw AIR. The data are represented as relative peak areas of major peaks for *p*-coumarate (*p*CA)-Ara and ferulate (FA)-Ara, previously identified by LC–MS as described by de Souza et al. [1]. *n* = 4; error bars ± SEM; significance of difference of transgenic lines from NT plants indicated if difference in means > least significant difference from ANOVA at *p* < 0.01 (*) and *p* < 0.001 (**)

In addition, the validation assay revealed that, under optimal pretreatment conditions, the biomasses of three distinct events showed more efficient saccharification, demonstrated by higher levels of glucose release compared to NT plants (Fig. 2a). As demonstrated, *SacBAHD01* RNAi lines 1, 2.2, and 2.4 presented an increase of 24%, 16%, and 11%, respectively, in biomass digestibility after *Organosolv* pretreatment and 48 h of enzymatic hydrolysis, compared to NT plants. Thus, these lines showing improved biomass digestibility were chosen to be submitted to a detailed cell-wall characterization.

### Cell-wall HCA contents and characterization of xylan in SacBAHD01 RNAi plants

Previously, we have shown that the silencing of *BAHD01* gene in the C4 model plant *Setaria viridis* caused a strong reduction of cell-wall-bound FA contents [1]. To verify if suppression of *BAHD01* gene in sugarcane would lead to similar effects, we analyzed transgenic lines that showed increased biomass digestibility to determine cell-wall-bound hydroxycinnamate (HCA) contents in the alcohol insoluble fraction (AIR) from the straw of these plants. It was observed that FA contents of transgenic lines decreased by 50% in *SacBAHD01* RNAi line 1 and *circa* 30% in the lines 2.2 and 2.4, compared to NT plants (Fig. 2b). It was also found that ester-linked *p*CA contents decreased *circa* 30% in cell walls of the lines 1 and 2.2, but the line 2.4 did not show statistically significant differences in total *p*CA contents, compared to NT plants (Fig. 2b).

Bound FA is ester-linked to arabinofuranosyl units attached to GAX of grass cell walls [36]. We found that silencing of *SvBAHD01* gene led to reductions in FA bound to arabinofuranosyl units (FA-Ara) in cell-wall tissues of *Setaria* [1]. Using mild acidolysis with trifluoroacetic acid (TFA) of AIR, we analyzed HCA-Ara contents in transgenic sugarcane, based on the methodology described in [1]. Using peak areas in ultraviolet absorbance spectra from this method, we observed reductions in FA-Ara contents of approximately 20% for *SacBAHD01* RNAi line 1 and 50% for lines 2.2 and 2.4 in the straw (Fig. 2c). *p*CA-Ara levels increased by 25% only in line 1, while lines 2.2 and 2.4 did not demonstrate significant changes compared with NT plants (Fig. 2c). In addition, we found no effects of *SacBAHD01* silencing on monosaccharides (arabinose, xylose, galactose, and glucose) and acetyl contents of AIR samples from sugarcane straw (Additional file 5: Table S2). The reduction of FA levels in the cell walls of sugarcane *SacBAHD01* silencing lines corroborated the results obtained from the suppression of this gene in the phylogenetically related plant *Setaria viridis*.

### Gel-state 2D-NMR characterization of cell walls in SacBAHD01 RNAi plants

**Fig. 3:**
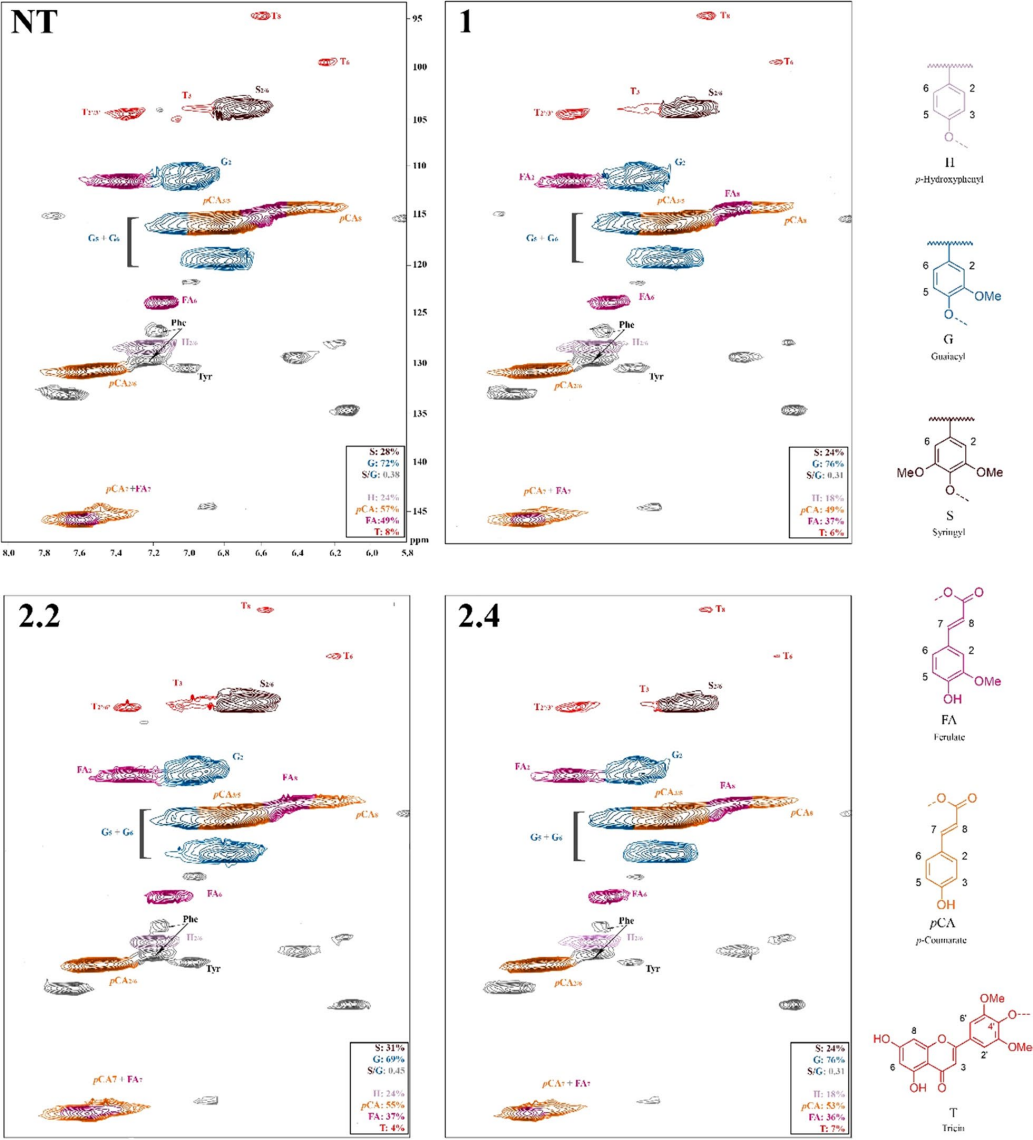
2D-NMR heteronuclear single-quantum coherence (HSQC) partial spectra of sugarcane straw from nontransformed (NT) plants and the three transgenic lines (1, 2.2, and 2.4). Color-coding of the contours matches that of the assigned structures; where contour overlap occurs, the colorization is only approximate. The analytical data are from volume integrals of correlation peaks representing reasonably well-resolved (except for H) C/H pairs in similar environments; thus they are from S_2/6_, G_2_, H_2/6_, FA_2_, *p*CA_2/6_, and T_2´6´_, with correction for those units that have two C/H pairs per unit. All relative integrals are on a G + S = 100% basis; H-units are over-quantified due to an overlapping peak from protein phenylalanine (Phe) units [38]

The total lignin content (Klason lignin) of sugarcane straw did not change between nontransformed (NT) and transgenic plants (Additional file 5: Table S2).To confirm the results obtained using biochemical analysis and to gain information on the overall aromatic composition of the unfractionated cell walls in the *SacBAHD01*-silenced plants, we performed gel-state 2D-NMR studies [37]. The spectral fingerprints presented in Fig. 3 demonstrate that FA levels decreased in transgenic lines compared to NT plants, corroborating the biochemical results. The decrease in FA contents in the cell walls of transgenic lines corresponded to 25%, as *p*CA contents decreased by 14.5%, 4%, and 7% in the lines 1, 2.2, and 2.4, respectively. It is worth mentioning that the 2D-NMR values presented here are on a lignin basis, and the integrals of small mobile components such as FA and *p*CA are variable in this methodology in relation to the immobile internal lignin units [1].

## Discussion

The cell walls of plants from the monocotyledon family Poaceae, which include grasses such as sugarcane, commonly have the presence of two hydroxycinnamates (HCAs), *p*-coumarate (*p*CA) and ferulate (FA) [10]. Cell-wall-bound FA mostly acylates glucuroarabinoxylans (GAX) present in the hemicellulose fraction of grasses [9], while most of the *p*CA acylates the lignin polymer [16]. For lignin, this is now known to occur by action of BAHD transferases acylating monolignol with FA or *p*CA-coenzyme A (CoA) acting as donors [38–42] and other studies suggest that analogous actions of similar but distinct BAHD transferases result in GAX acylation by *p*CA and FA transferases [1, 19–21, 43]. The crosslinks promoted by the acylation of GAX by FA contribute for the inhibition of biomass saccharification by tightly binding the polysaccharide substrate to the nondigestible lignin. Therefore, decreasing the FA levels or FA-mediated crosslinking of grass biomass has been considered a promising target for increasing digestibility [17]. In fact, we have shown that the silencing of *BAHD01* gene in green foxtail (*Setaria viridis*), a model plant for C4 grasses [22], caused large decreases in cell-wall feruloylation, thereby increasing biomass saccharification [1]. Here, we characterized the *BAHD acyl*-*CoA transferase* gene family in sugarcane (*Saccharum* spp.) and, using an RNAi approach, suppressed the ortholog of *Setaria BAHD01* in this crop.

We were able to find six members of the candidate clade of *BAHD* genes [18] in the sugarcane genome (Fig. 1a). The number of *BAHD* genes found in the sugarcane genome appeared to be much less compared to phylogenetically related plants such as rice (10), maize (9), sorghum (9), *Brachypodium* (9) and *Setaria* (10). However, modern cultivars of sugarcane have an extremely complex genome, derived from interspecific hybridization, as is the case for the variety used in the present study (SP80-3280). Sugarcane elite cultivars might have up to 130 chromosomes, distributed among ~ 12 homo (eo)logous groups [44], with a total genome size reaching 10 Gbp [45], and such complex genome structure hinders genome sequencing, assembly and annotation. Our phylogenetic analysis is based on the recently published draft genome sequencing of the sugarcane hybrid SP80-3280 [32] and R570 variety [33], despite the long reads used in assembly of this genome, we cannot exclude the possibility that some gene sequences could be missing. Thus, it is plausible that sugarcane might have other members of the BAHD clade, which we could not identify in our analysis.

The *BAHD* genes identified in sugarcane were all expressed in leaf tissues from two different developmental stages, with *SacBAHD01* and *SacBAHD05* presenting the highest levels of expression (Fig. 1b). *SacBAHD01*, implicated in FA incorporation in the cell wall of grasses [1], was highly expressed in young leaves (3-month-old plants) of sugarcane, while its transcript levels decreased ~ 5-fold in mature leaves (8-month-old plants). In contrast, the other *SacBAHD* genes identified (03, 05, and 09) were all expressed in young and mature leaves, but their transcript levels did not drastically change during these different developmental stages. These results are in agreement with previous studies on *Brachypodium BAHD* genes, where *BdBAHD01*, *05,* and *09* were highly expressed in vegetative tissues, with only *BdBAHD05* and *09* being expressed in later stages of reproductive development [46]. From these four *BAHD* genes identified in sugarcane, *BAHD01*, *05*, and *09* have a putative assigned role in other grasses. BAHD01 and BAHD05 proteins are potential candidates for feruloylation of GAX in *Setaria* and *Brachypodium*, respectively, while orthologs of *BAHD09* are candidates for the addition of *p*CA to monolignols, being classified as a BAHD PMT (*p*-coumaroyl monolignol transferase) [40, 41, 43]. As feruloylation of GAX tends to occur in both primary and secondary grass cell walls, which are biosynthesized during the early stages of development [46], it is not surprising to see the high expression level of *SacBAHD01* in young leaves of sugarcane (Fig. 1b). These results reinforce that *BAHD01* is a putative candidate for feruloylation of GAX in grasses.

To test if *BAHD01* silencing would have any effect on biomass digestibility, we used sugarcane straw from transgenic BAHD01 RNAi lines, comparing them to nontransformed plants. We used the sugarcane straw as biomass for the studies on digestibility because this material does not require processing, as is necessary for the production of bagasse, which is generated after the juice extraction from the stalks.

The utilization of less recalcitrant materials for bioethanol production is not sufficient to ensure maximum glucose release from the biomass. Therefore, biomass pretreatments are fundamental in industrial processes. Nevertheless, the pretreatment followed by enzymatic hydrolysis of the biomass consist in a large proportion of the costs involved in the biorefinery process, being a bottleneck for the adaptation of sugarcane mills to produce cellulosic ethanol [47]. For the pretreatments available, the *Organosolv* process using ethanol as solvent appears to be the most promising for many reasons: (*i*) low cost and ease of recovery; (*ii*) low energy requirement for solvent recovery; (*iii*) fully miscible in water; (*iv*) low toxicity. Usually, the *Organosolv* treatment of the biomass is preceded by addition of acids, which act as catalysts for the rupture of the lignin-carbohydrate complex [31, 48]. Our results demonstrated that silencing of *BAHD01* in sugarcane resulted in the improvement of biomass saccharification after *Organosolv* pretreatment, suggesting a decrease in biomass recalcitrance (Fig. 2a). However there was no simple relationship between variation in FA or FA-Ara reduction between lines and benefit to saccharification (Fig. 2); this was also the case for the two lines of *Setaria* we studied in detail [1]. For instance, transgenic line 1 demonstrated highest levels of saccharification, but total FA and FA-Ara levels in this line were higher compared to lines 2.2 and 2.4 (Fig. 2). Therefore, it is possible that other factors are involved in determining saccharification efficiency. In fact, two major characteristics of cell wall—the cellulose crystalline index (CrI) and the degree of polymerization (DP) of β-1,4-glucans—have been considered essential to negatively affect biomass digestibility under various pretreatments in different species, including sugarcane [25, 26, 49, 50]. In addition, recent studies have suggested that the arabinose (Ara) substitution degree of xylans could reduce cellulose crystallinity for positively affecting biomass enzymatic digestibility under chemical pretreatments in sugarcane and other grasses [25, 51, 52]. In our studies, transgenic line 1 presented increased levels of *p*CA-Ara (Fig. 2c), and it is tempting to speculate that the highest levels of digestibility found for its biomass could be related to decreased CrI due to the higher degree of substitution caused by *p*CA-Ara. However, further investigations on cell-wall CrI and cellulose DP of our transgenic lines are required and are currently being investigated.

In the present study, we did not use catalysts before ethanol treatment, but we obtained high levels of glucose release under optimal conditions of pretreatment and enzymatic hydrolysis (Table 1; Fig. 2a). These results suggest that biomass from sugarcane *BAHD01*-silenced lines is a suitable material for the generation of cellulosic ethanol in industrial scale, as the increase of fermentable glucose levels reached up to 24% in line 1, compared to nontransformed plants. These high levels of saccharification were achieved under low ethanol concentration and temperature (30% v/v, 180 °C, respectively), and in a relatively short period of time (120 min).

The cell-wall characterization of sugarcane transgenic lines demonstrated that silencing of *BAHD01* decreased the levels of FA and FA-Ara, with no significant changes in total lignin or monosaccharide composition (Figs. 2, 3; Additional file 5: Table S2). These results were also found when *BAHD01* was silenced in *Setaria*, indicating that the species is in fact a suitable experimental model for grasses.

Other approaches have achieved increases in saccharification yields from sugarcane residues by silencing or knock-out of genes responsible for lignin synthesis [53, 54]. In contrast to our strategy, this alternative approach results in lower lignin which can be a valuable feedstock in biorefining; in our approach, less FA-mediated crosslinking could enable greater ease of separation of lignin from polysaccharide. Further work with field-grown sugarcane using different technologies to utilize the bagasse and straw are required to determine the best approaches. In addition, it is known that aromatic carboxylic acids such as FA are strong inhibitors of microbial growth [55]. Larsson et al. [56] demonstrated that ferulic acid strongly inhibited *S. cerevisiae* growth at very low concentrations (1.0 mM). As the concentration of FA in sugarcane bagasse hydrolysates can reach 1.1 mM [57], our strategy of decreasing FA levels is advantageous for industrial processes since it is possible to diminish inhibitory effects during the fermentation process.

In summary, the silencing of a BAHD acyl transferase gene in sugarcane decreased the levels of FA in cell walls, increasing the saccharification levels of its biomass, leading to great opportunities to develop new elite cultivars for bioethanol production.

## Conclusions

The silencing of *SacBAHD01* gene and subsequent decrease of cell-wall ferulate contents indicate a promising biotechnological approach for improving the suitability of sugarcane residues for cellulosic ethanol production. In addition, the *Organosolv* pretreatment of the genetically modified biomass and the optimal conditions for the enzymatic hydrolysis presented here might be incorporated in the sugarcane industry for bioethanol production.

## Methods

### Phylogenetic analysis

Whole genome sequences of SP80-3280 and R570 sugarcane varieties were downloaded from http://bce.bioetanol.cnpem.br/sugarcanegenome and http://sugarcane-genome.cirad.fr/ to identify potential BAHD protein sequences, respectively. Sequences from previously identified BAHD proteins in *Brachypodium*, rice, maize, sorghum, and *Setaria* [1, 18, 46] were used as query to search for the BAHD sequences in the sugarcane genome database using tblastn, with *e*-value of 10^−10^ as threshold, to identify sequences with high similarity. A custom script was then developed and executed to remove redundant sequence and retrieve the similar sequence regions from sugarcane genome. The phylogenetic analysis was performed after the alignment of the protein sequences using MUSCLE, and the phylogenetic tree was estimated by a maximum-likelihood method using FastTree 2.1.5 program [58]. The tree was visualized with the iTOL software (https://itol.embl.de/).

### Expression analysis of *SacBAHD* genes in sugarcane leaves

Top leaves (a pool of four fully expanded leaves) of 3- and 8-month-old SP80-3280 sugarcane variety were collected for expression analysis of the identified *SacBAHD* genes (*SacBAHD01*, *03*, *05* and *09*) from three different plants. Total RNA was isolated using TRIzol reagent (Invitrogen, Grand Island, NY) and treated with RNaseFree RQ1 DNase (Promega, San Luis Obispo, CA) according to the manufacturer’s instructions. cDNA was synthesized from one µg of RNA using SuperScript^®^ III kit (Invitrogen). The expression level was normalized against the sugarcane Glyceraldehyde 3-phosphate dehydrogenase (GAPDH) and Elongation factor 1-alpha (EF1) genes by qRT-PCR. The reactions were performed with SYBR Green Master Mix (Applied Biosystems) under the following conditions: 95 °C for 3 min denaturation, 40 cycles at 95 °C for 10 s, and 58 °C for 45 s. Amplification specificity was verified by melt curve analysis from 55 to 95 °C. *SacBAHD* expression levels were calculated using the 2^−∆∆Ct^ method [59]. The primers’ sequences used for GAPDH (CA254672), EF1 (XXX), and *SacBAHDs (*SacBAHD1: MK614571; SacBAHD3: MK614570; SacBAHD5: MK614573; SacBAHD9: MK614572) amplifications are listed in Additional file 6: Table S3.

### Generation of transgenic sugarcane plants

After the identification of *BAHD01* gene in the sugarcane genome and verification of its expression in sugarcane leaves, we selected a 413 bp sequence to design a construct containing inverted repeats of this sequence flanking the maize *Adh1* intron, in order to silence *SacBAHD01* gene (Additional file 1: Fig. S1a). In this construct, synthesized by DNA Cloning Service (Hamburg, Germany), the *SacBAHD01* RNAi cassette is under control of the maize ubiquitin promoter, and the *bar* gene used as selectable marker is under control of the rice actin 1 promoter. This construct was used to transform embryogenic callus of SP80-3280 sugarcane variety, following our published protocol [60].

### Molecular analysis of transgenic plants

Genomic DNA from regenerated sugarcane plantlets resistant to the herbicide bialaphos was extracted using a modified CTAB method [61]. The gene insertion was confirmed by conventional PCR using specific primers designed for the *bar* gene amplification (Additional file 6: Table S3). The candidate transgenic events were submitted to analysis by real-time PCR of the target gene *SacBAHD01*, using GAPDH and EF1-α as reference for normalization of transcripts as described above. Plants from independent transgenic events demonstrating the highest levels of *SacBAHD01* suppression were vegetatively propagated, and leaves from 8-month-old plants were collected for confirmation of the stability of *BAHD01* suppression. Nontransformed (NT) plants, which consist of plants from cell tissue culture, were included in all studies. After confirmation, the straw of these plants was collected for further analysis.

### *Organosolv* pretreatment and enzymatic hydrolysis

Sugarcane leaves (four fully expanded top leaves) and stem tops (around 30 cm) of 12-month-old plants (straw) were collected for pretreatment. The straw is composed of approximately 60% leaves and 40% stem tops. The straw was chopped, and the samples were pretreated using *Organosolv* process and ethanol as organic solvent. The pretreatment was conducted in 19-L batch reactor PARR 4555 using 3 L of solvent and 5 g (dry weight) of each biomass placed in baskets (nontransformed (NT) plants, and five independent *SacBAHD01* RNAi lines). The starting point of the residence time was considered as temperature in the reactor reached 180 °C. Different concentrations of ethanol (ranging from 30 to 100%) and residence times (ranging from 0 to 240 min) were used in our studies.

In order to evaluate and optimize the effects of ethanol concentration (*X*1) and residence time (*X*2) in enzymatic saccharification, after pretreatment, we applied the Response Surface Methodology (RSM) based on Central Composite Design (CCD). RSM is a tool based on statistical theory that provides reliable information about the process, reducing the number of experiments or repetitions and improving the quality of the information. A CCD design is a specific type of experimental design that contains a factorial design with center points, which is augmented by a group of axial points that allow estimation of curvature. When the distance from the center of the design region to a factorial point is ± 1 unit for each factor, the distance from the center to axial point, considering the rotatability of factorial plan, is |*α*| = (2^k^)^1/4^ > 1, where k is a number of factors. Then, axial points represent two new extreme levels totalizing five levels (− *α*, − 1, 0, + 1, + *α*) for each factor. This technique is also useful to analyze factors simultaneously and optimize more than one response at a time [34, 35]. This experimental design considered two independent variables (*X*1 and *X*2), three replicates at the center point, alpha for rotatability of 1.4, resp experiments completely randomized. Glucose concentrations released after 6 and 48 h of enzymatic hydrolysis, considering four replicates, were used as the responses in CCD. Table 1 shows coded and original values used in CCD experiments. The original values have to be coded to keep the proportionality between the effects of the variables. When working with coded values, these are independent of the variables values and vary steadily from − 1.41 to + 1.41. The value of the center point (level 0) is determined considering the average distance between levels − 1.41 and + 1.41. Levels + 1 and − 1 are proportional to the levels previously determined.

For enzymatic hydrolysis, pretreated samples were dried in an oven overnight at 40 °C. Enzymatic saccharification assays were performed in 2 mL tubes with 5% (w/v) dry biomass in 100 mM citrate buffer, pH 5.0. The cellulase/hemicellulase cocktail Cellic^®^ CTec 3 (Novozymes, Lyngby, Denmark) was added at the desired filter paper activity units FPU/g biomass (ranging from 3 to 30 FPU). The reaction was incubated in a Thermomixer microplate incubator (Eppendorf, Germany) operated at 50 °C and agitation speed of 800 rpm. Samples were withdrawn after 6 and 48 h, followed by centrifugation at 10,000*g* for 15 min. Sugars in enzyme hydrolysates were analyzed by high-performance liquid chromatography system HPLC (Agilent, Palo Alto, CA-USA), equipped with a refractive index detector and Aminex HPX-87H ion exchange column (Bio-Rad, Hercules, CA-USA). The mobile phase contained 5 mM H_2_SO_4_, at a flow rate of 0.6 mL min^−1^, heated at 45 °C. The experiments were carried out in four technical replicates, and the statistics were applied based on the data collected from three different plants.

The statistical analysis was employed to verify significant differences between NT and independent transgenic plants after hydrolysis, using the Tukey test at 95% probability. The software Statistica™ 12.0 (Statsoft, Palo Alto, Ca-USA) was used for the design of experiments, regression, graphical analyses, and analyses of variance. The second-degree polynomials (Eq. 1) were calculated to estimate the response of the dependent variables, *X*1 and *X*2. Significance level was considered to be of 90%.

1 $$Y \, = \, b0 \, + \, b1X1 \, + \, b2X2 \, + \, b11X1^{2} + \, b22X2^{2} + \, b12X1X2,$$

where *Y* is the predicted response; *X*1 is organic solvent concentration in encoded value; *X*2 is residence time in encoded value; *b*0 is offset term; *b*1, *b*2 are linear effects; *b*11, *b*22 are squared effects; and *b*12 are the interaction terms. Canonical analysis and eigenvalues were used to locate the stationary point of the responses and to determine whether it represents a maximum point. To validate the model predictions, an additional pretreatment was carried out under conditions predicted by the model, followed by enzymatic hydrolysis.

### Quantitation of cell-wall-bound hydroxycinnamate (HCA) content and determination of HCA conjugates released by mild acidolysis

The content of the ester-linked cell-wall-bound hydroxycinnamates (*p*-coumarate and ferulate) was determined essentially as described [62], using the straw of 12-month-old nontransformed (NT) plants and sugarcane transgenic lines 1, 2.2, and 2.4 as samples. First, the biomass was chopped and sieved to obtain a fine powder, and then freeze-dried. The determination of HCA conjugates was performed as described by de Souza et al. using 10 mg freeze-dried ground tissue [1]. Briefly, the alcohol-insoluble residue (AIR, prepared using extractions as described for cell-wall-bound HCA) was treated with 1.2 mL 50 mM trifluoroacetic acid for 4 h at 99 °C with agitation at 750 rpm. After centrifugation for 10 min at 16,000*g* 2× 500 μL aliquots of supernatant were freeze-dried. The pellet was washed twice with water and freeze-dried. Released HCA-conjugates from one 500 μL aliquot of supernatant were dissolved in 250 µL 50% methanol, 0.1% formic acid, and 10 μL separated as for cell-wall-bound HCA except using a binary gradient with methanol (solvent A) and 0.1% formic acid (solvent B), under the following conditions: isocratic 100% B, 0–1 min; linear 100% to 0% B, 1–21 min; isocratic 0% B, 21–23 min; and linear 0% to 100% B, 23–28 min at a flow rate of 1 mL min^−1^. The results for HCA-Arabinose (HCA-Ara) were expressed as relative peak areas (absorbance at 280 nm) corresponding to major peaks for *p*-coumarate (*p*CA)-Ara and ferulate (FA)-Ara, previously identified by LC–MS [1]. The statistical analysis was performed using ANOVA at *p* < 0.05 (*) and *p* < 0.001 (**), with *n* = 3.

### Cell-wall characterization by solution-state two-dimensional NMR

Sugarcane cell walls were characterized using solution-state 2D NMR according to procedure described by [37]. Aliquots of approximately 500 mg (in triplicate) of freeze-dried ground tissue (straw) of each biological replicate from NT control and events 1, 2.2, and 2.4 were weighed and extracted overnight (minimum of 8 h) with a mixture acetone/water (95:5 v/v) on a Soxhlet apparatus (~ 70 °C). The extract-free samples were oven-dried at 50 °C for 48 h. The dried extract-free plant material was submitted to a ball-milling procedure. Aliquots of 200 mg of each sample were milled in 20 mL jars with 10 × 10 mm ball bearings in a Fritsch^®^ Planetary micro mill Pulverisette 7 premium line equipment, according to following milling protocol: 5 × 5 min with 5 min pauses in between. After the ball-milling procedure, the preparation for NMR analysis for all samples was carried out according to [37] for gelling samples without derivatization, and their conditions used for acquisition of the NMR spectra and processing. The acquisition of the NMR spectra were performed at Laboratory of Nuclear Magnetic Resonance of Federal University of Sao Carlos (São Carlos-SP/Brazil), on a 600 MHz Bruker^®^ AVANCE III spectrometer system equipped with a 5 mm TCI cryoprobe with ATMA^®^ (Automatic Tuning and Matching).

### Total lignin content and monosaccharide composition

The total lignin content (Klason lignin) and the monosaccharide and acetyl composition of AIR samples were determined following the protocol described by the National Renewable Energy Laboratory of the US Department of Energy [63].

## Additional files

**Additional file 1: Figure S1.** BAHD01 RNAi cassette and alignment of region targeted by the cassette.

**Additional file 2: Figure S2.** Expression analysis and silencing levels of transgenic *SacBAHD01* RNAi lines.

**Additional file 3: Figure S3.** Enzymatic hydrolysis (saccharification) of wild-type sugarcane straw after *Organosolv* pretreatment.

**Additional file 4: Table S1.** Spreadsheet containing quadratic model equations, in coded values, for 6 and 48 h enzymatic hydrolysis for events and NT plants.

**Additional file 5: Table S2.** Determination of Klason lignin, monosaccharide and acetyl composition of AIR from straw of control and *SacBAHD01* RNAi transgenic plants.

**Additional file 6: Table S3.** Primers used in this study.

## Data Availability

The authors ensure the availability of supporting data.
