# Correction to: Silencing of a BAHD acyltransferase in sugarcane increases biomass digestibility

**DOI:** 10.1186/s13068-019-1482-z

**Published:** 2019-06-08

**Authors:** Wagner Rodrigo de Souza, Thályta Fraga Pacheco, Karoline Estefani Duarte, Bruno Leite Sampaio, Patrícia Abrão de Oliveira Molinari, Polyana Kelly Martins, Thaís Ribeiro Santiago, Eduardo Fernandes Formighieri, Felipe Vinecky, Ana Paula Ribeiro, Bárbara Andrade Dias Brito da Cunha, Adilson Kenji Kobayashi, Rowan Andrew Craig Mitchell, Dasciana de Sousa Rodrigues Gambetta, Hugo Bruno Correa Molinari

**Affiliations:** 1 Genetics and Biotechnology Laboratory, Embrapa Agroenergy (CNPAE), Brasília, DF 70770-901 Brazil; 2 Centre of Natural Sciences and Humanities, Federal University of ABC, São Bernardo do Campo, SP 09606-045 Brazil; 3 Plant Sciences, Rothamsted Research, Harpenden, Hertfordshire AL5 2JQ UK

## Correction to: Biotechnol Biofuels (2019) 12:111 10.1186/s13068-019-1450-7

After the publication of the article [1], it was brought to our attention that the GenBank Accession number for the EF1 is missing in Methods section. Please find the accession number in the erratum below.

### Expression analysis of SacBAHD genes in sugarcane leaves

Top leaves (a pool of four fully expanded leaves) of 3- and 8-month-old SP80-3280 sugarcane variety were collected for expression analysis of the identified SacBAHD genes (SacBAHD01, 03, 05 and 09) from three different plants. Total RNA was isolated using TRIzol reagent (Invitrogen, Grand Island, NY) and treated with RNaseFree RQ1 DNase (Promega, San Luis Obispo, CA) according to the manufacturer’s instructions. cDNA was synthesized from one µg of RNA using SuperScript^®^ III kit (Invitrogen). The expression level was normalized against the sugarcane Glyceraldehyde 3-phosphate dehydrogenase (GAPDH) and Elongation factor 1-alpha (EF1) genes by qRT-PCR. The reactions were performed with SYBR Green Master Mix (Applied Biosystems) under the following conditions: 95 °C for 3 min denaturation, 40 cycles at 95 °C for 10 s, and 58 °C for 45 s. Amplification specificity was verified by melt curve analysis from 55 to 95 °C. SacBAHD expression levels were calculated using the 2^−∆∆Ct^ method [59]. The primers’ sequences used for GAPDH (CA254672), EF1 (EF581011.1), and SacBAHDs (SacBAHD1: MK614571; SacBAHD3: MK614570; SacBAHD5: MK614573; SacBAHD9: MK614572) amplifications are listed in Additional file 6: Table S3.
