# Supplementary material for: Silencing of a BAHD acyltransferase in sugarcane increases biomass digestibility
Source: Biotechnol Biofuels. 2019 May 6;12:111. doi: 10.1186/s13068-019-1450-7 (PMC6501328; doi:10.1186/s13068-019-1450-7)
Supplement: Supplementary file 1 — Additional file 1: Figure S1. BAHD01 RNAi cassette and alignment of region targeted by the cassette. [file 13068_2019_1450_MOESM1_ESM.docx]

**Supplemental Figure 1.**

**
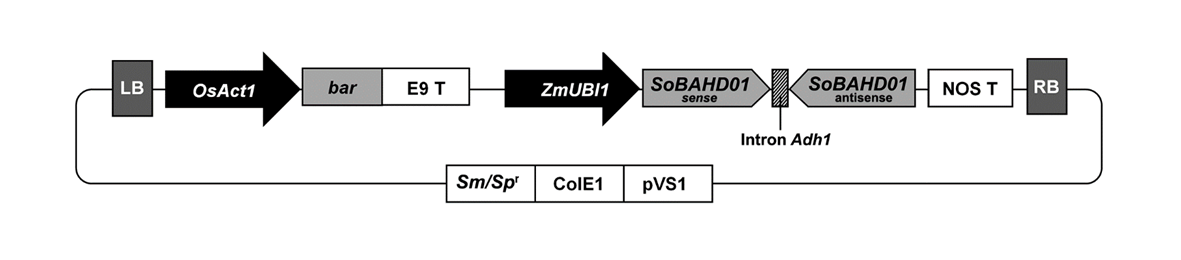
(a)**

**
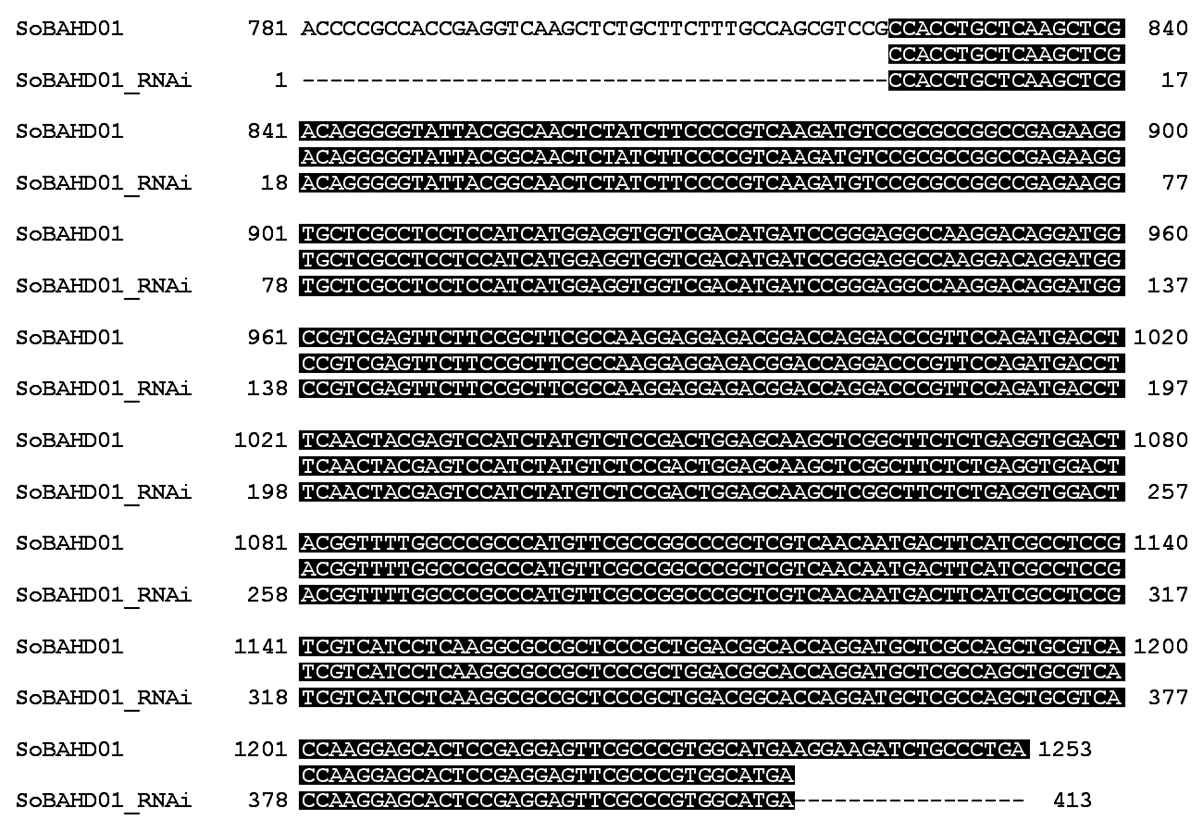
(b)**

**Supplemental Figure 1. *BAHD01* RNAi cassette and alignment of region targeted by the cassette. (a)** Sac*BAHD01* suppression is given by expression of an RNAi hairpin under control of a constitutive maize ubiquitin promoter flanking the maize *Adh1* intron. The *bar* gene used as selectable marker, conferring resistance to the herbicide bialaphos, is under control of the rice actin 1 promoter (Os*Act1*). **(b)** Alignment of region targeted by SacBAHD01 RNAi (SacBAHD01_ RNAi) and sugarcane *BAHD01* gene (SacBAHD01).
