# Supplementary material for: Silencing of a BAHD acyltransferase in sugarcane increases biomass digestibility
Source: Biotechnol Biofuels. 2019 May 6;12:111. doi: 10.1186/s13068-019-1450-7 (PMC6501328; doi:10.1186/s13068-019-1450-7)
Supplement: Supplementary file 2 — Additional file 2: Figure S2. Expression analysis and silencing levels of transgenic SacBAHD01 RNAi lines. [file 13068_2019_1450_MOESM2_ESM.docx]

**Supplemental Figure 2.**

**(a)**

**
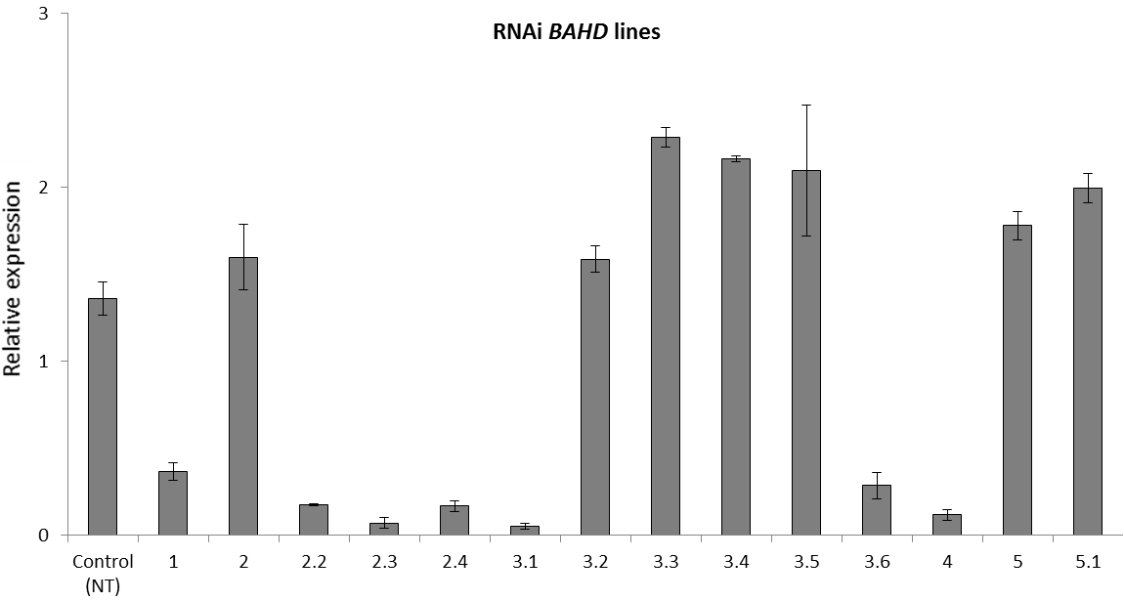
**

**(b)**

**
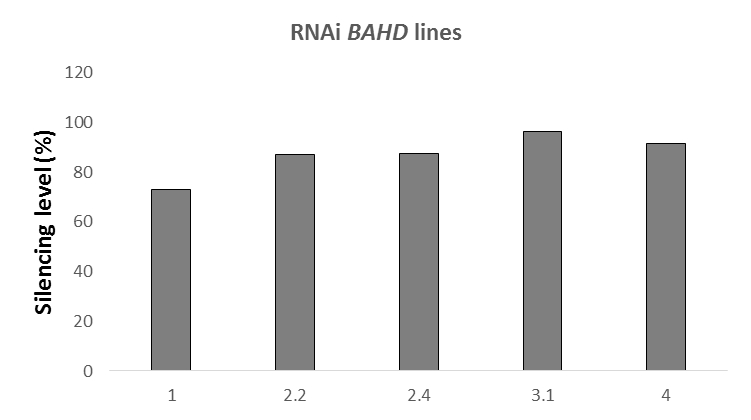
**

**Supplemental Figure 2. Expression analysis and silencing levels of transgenic SacBAHD01 RNAi lines. (a)** Real-time qPCR analysis of 14 independent events of transgenic sugarcane transformed with SacBAHD01 RNAi cassette. Leaves of 3-month-old plants were used for expression analysis (*n* = 5; error bars ± SEM). **(b)** Silencing levels of the most BAHD01 suppressed lines (events 1, 2.2, 2.4, 3.1 and 4), as observed during RT-qPCR analysis. The silencing levels were represented as the percentage of silencing in transgenic plants compared to non-transformed plants (NT).
