# Supplementary material for: Silencing of a BAHD acyltransferase in sugarcane increases biomass digestibility
Source: Biotechnol Biofuels. 2019 May 6;12:111. doi: 10.1186/s13068-019-1450-7 (PMC6501328; doi:10.1186/s13068-019-1450-7)
Supplement: Supplementary file 3 — Additional file 3: Figure S3. Enzymatic hydrolysis (saccharification) of wild-type sugarcane straw after Organosolv pretreatment. [file 13068_2019_1450_MOESM3_ESM.docx]

**Figure S3.**


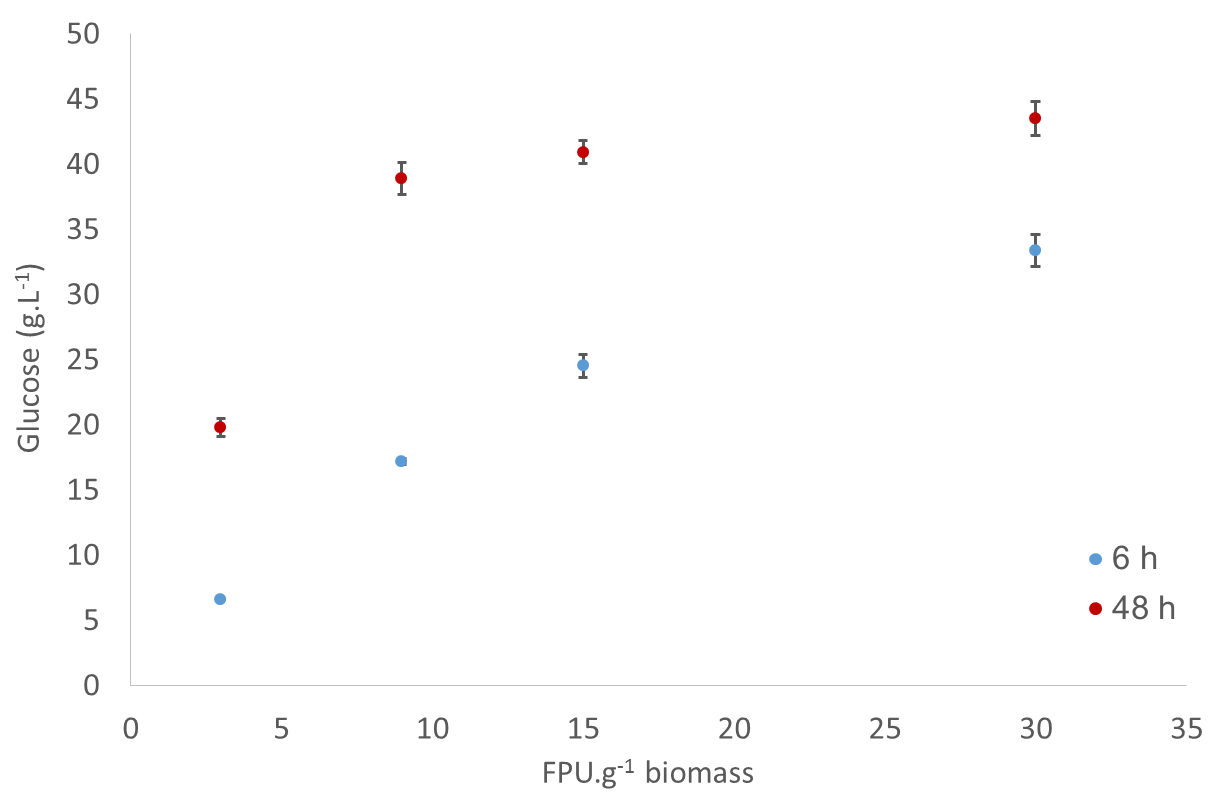


**Figure S3.** Enzymatic hydrolysis (saccharification) of wild-type sugarcane straw after *Organosolv* pretreatment using increasing enzymatic loading during 6 and 48 h. FPU: filter paper units.
