# Supplementary material for: Silencing of a BAHD acyltransferase in sugarcane increases biomass digestibility
Source: Biotechnol Biofuels. 2019 May 6;12:111. doi: 10.1186/s13068-019-1450-7 (PMC6501328; doi:10.1186/s13068-019-1450-7)
Supplement: Supplementary file 5 — Additional file 5: Table S2. Determination of Klason lignin, monosaccharide and acetyl composition of AIR from straw of control and SacBAHD01 RNAi transgenic plants. [file 13068_2019_1450_MOESM5_ESM.docx]

**Table S2.** Klason lignin, monosaccharide and acetyl composition of AIR from non-pretreated straw of control (NT, non-transformed plants) and SacBAHD01 RNAi transgenic plants descended from events (Ev.) 1, 2.2 and 2.4. Values are means (%) ± SEM from five different replicates**^*^**.

| **Sample** | **Monosaccharide and acetyl composition of AIR (Mean ± SE)** | | | | | |
| --- | --- | --- | --- | --- | --- | --- |
|  | **Lignin** | **Arabinose** | **Galactose** | **Glucose** | **Xylose** | **Acetyl** |
| ***NT*** | 16.86 ± 0.07 | 2.61 ± 0.03 | 0.72 ± 0.01 | 30.70 ± 0.13 | 16.91 ± 0.11 | 2.60 ± 0.05 |
| ***Ev. 1*** | 16.60 ± 0.23 | 2.61 ± 0.04 | 0.68 ± 0.02 | 30.93 ± 0.50 | 16.81 ± 0.31 | 2.54 ± 0.03 |
| ***Ev. 2.2*** | 16.91 ± 0.32 | 2.90 ± 0.07 | 0.67 ± 0.01 | 31.40 ± 0.31 | 16.42 ± 0.22 | 2.68 ± 0.04 |
| ***Ev.2.4*** | 16.49 ± 0.09 | 2.86 ± 0.08 | 0.64 ± 0.03 | 30.93 ± 0.48 | 16.27 ± 0.32 | 2.71 ± 0.04 |

**^*^**Mean values represent the percentage of structural carbohydrates in the cell wall (raw material). The extractives represented ~ 30% of the non-pretreated biomass and proteins and inorganic components represented ~ 6% and 2%, respectively, in all samples examined (not included in the Table).
