# Supplementary material for: Silencing of a BAHD acyltransferase in sugarcane increases biomass digestibility
Source: Biotechnol Biofuels. 2019 May 6;12:111. doi: 10.1186/s13068-019-1450-7 (PMC6501328; doi:10.1186/s13068-019-1450-7)
Supplement: Supplementary file 6 — Additional file 6: Table S3. Primers used in this study. [file 13068_2019_1450_MOESM6_ESM.docx]

**Table S3:** Primers used in this study.

| **Gene** | **Primer name** | **Sense** | **Sequence** | **ID*** |
| --- | --- | --- | --- | --- |
| SacBAHD1 | SacBAHD1 | F | AGTCCATCTATGTCTCCGACTG | MK614571 |
|  |  | R | GGAGGCGATGAAGTCATTGTT |  |
| SacBAHD3 | SacBAHD3 | F | CCAAGGACGACCACTACAAC | MK614570 |
|  |  | R | TTGAGCGTGAAGACGTACC |  |
| SacBAHD5 | SacBAHD5 | F | GATCTCTCTGGACAGCATCAAG | MK614573 |
|  |  | R | CGCGGCATTTGAAGATTATGG |  |
| SacBAHD9 | SacBAHD9 | R | TCTCCTACCACCCGTTCAT | MK614572 |
|  |  | F | GTTCATCTGCTCCTGGAACTC |  |
| Glyceraldehyde 3-phosphate dehydrogenase | SacGAPDH | R | GTGTCAACGAGAAGGAGTACAA | CA254672 |
|  |  | F | GTCATCAGACCCTCAACGATAC |  |
| Elongation factor 1-alpha | SacEF1 | F | TTTCACACTTGGAGTGAAGCAGAT | EF581011.1 |
|  |  | R | GACTTCCTTCACAATCTCATCATAA |  |
| *bar* | BAR | F | GTCAACCACTACATCGAGACAA | KF780168.1 |
|  |  | R | TCAGCAGGTGGGTGTAGA |  |

*Sequence IDs from GenBank (NCBI).
